# 

**Published:** 1885-07

**Authors:** 


					﻿VoK VI.	1885.	No. 7.
THE
IntatndentPraetitoer:
> MONTHLY
DEVOTED TO
DENTAL AND ORAL SCIENCE.
W. C. BARRETT, M. D„ D. D. S.,
EDITOR.
The Hew York Dsétal Journal Association,
PUBLISHERS,
No, 35 West 46 th Street, New York,
AND
11 W. Chippewa St., Hu Halo, N. Y.
$2.50 Per Annum in Advance, .... Single Copies, 25 Cents.
Entered at the Post-Office, Buffalo, N. Y., as Second-Class matter.
				

